# Helical X-ray phase-contrast computed tomography without phase stepping

**DOI:** 10.1038/srep23953

**Published:** 2016-04-07

**Authors:** M. Marschner, M. Willner, G. Potdevin, A. Fehringer, P. B. Noël, F. Pfeiffer, J. Herzen

**Affiliations:** 1 Lehrstuhl für Biomedizinische Physik, Physik-Department & Institut für Medizintechnik, Technische Universität München, 85748 Garching, Germany; 2 Institut für diagnostische und interventionelle Radiologie, Klinikum rechts der Isar, Technische Universität München, 81675 München, Germany

## Abstract

X-ray phase-contrast computed tomography (PCCT) using grating interferometry provides enhanced soft-tissue contrast. The possibility to use standard polychromatic laboratory sources enables an implementation into a clinical setting. Thus, PCCT has gained significant attention in recent years. However, phase-contrast CT scans still require significantly increased measurement times in comparison to conventional attenuation-based CT imaging. This is mainly due to a time-consuming stepping of a grating, which is necessary for an accurate retrieval of the phase information. In this paper, we demonstrate a novel scan technique, which directly allows the determination of the phase signal without a phase-stepping procedure. The presented work is based on moiré fringe scanning, which allows fast data acquisition in radiographic applications such as mammography or in-line product analysis. Here, we demonstrate its extension to tomography enabling a continuous helical sample rotation as routinely performed in clinical CT systems. Compared to standard phase-stepping techniques, the proposed helical fringe-scanning procedure enables faster measurements, an extended field of view and relaxes the stability requirements of the system, since the gratings remain stationary. Finally, our approach exceeds previously introduced methods by not relying on spatial interpolation to acquire the phase-contrast signal.

X-ray computed tomography is a widely utilized tool for medical and industrial applications. Phase-sensitive X-ray techniques are an interesting, more sensitive method for visualizing details with similar density, as X-rays are phase-shifted significantly more than absorbed. Consequently, improved image quality and contrast can be achieved when utilizing the phase information of an object^1^. In the past, several methods for phase-contrast imaging have been developed, but most of them are restrained to highly brilliant X-ray sources. With the recent introduction of grating interferometry, which relies on transmission gratings with μm-size periods, high-sensitivity phase measurements are possible with laboratory sources^2^,^3^. The potential benefit arising from using grating interferometry for biomedical imaging has been verified by several investigators^4^,^5^.

The utilized interferometer is a so-called Talbot-Lau interferometer as shown in Fig. 1(a), which consists of three gratings. The first grating (*G*_0_) is an absorber grating, which is placed directly behind the source and provides transverse beam coherence. The second grating (*G*_1_), which is placed close to the sample to enhance sensitivity, is a phase grating that imposes a periodic phase modulation onto the X-ray beam. This creates periodic intensity modulations downstream of the beam. A phase-shifting (refractive) sample in the beam leads to a displacement of this pattern. The third grating (*G*_2_) is an absorber grating with a period matching the intensity pattern and is installed to analyse this pattern. It cannot be resolved directly with conventional detectors because the period of the gratings and the interference patterns have to be small to resolve even slight phase shifts. The prevailing measurement procedure relies on translation of one of the gratings. The sample is recorded multiple times with different relative positions of the gratings. These images can be combined to phase-stepping curves for each pixel^3^,^6^. The information related to absorption, refraction and scattering strength can then be extracted by Fourier analysis or a least-squares fit of these curves. This so-called phase-stepping procedure is a limiting factor in the reduction of the overall acquisition time, because it limits the rotation speed of the sample or the gantry. Further, a precise translation of the grating is required, which implies strict stability conditions of the system. In many applications, fast image acquisition is of great importance, but is, however, not realized with current phase-contrast CT.

Recently, several procedures were proposed to circumvent the need of phase stepping at each rotation step.

While interlaced phase stepping^7^ allows a continuous rotation by combining rotation step and phase step, a grating still needs to be moved. This implies the same stability conditions as any other phase-stepping procedure. Additionally, the translation of the grating is limited with respect to speed and accuracy and therefore restricts improvements in rotation speed and image quality.

In comparison, single-shot fringe analysis is an approach that can retrieve the phase information without a stepping procedure^8^,^9^. It is also possible to fabricate gratings in a way that they intrinsically feature different stepping positions for different detector lines in order to obtain the phase information from only one image^10^. However, the major shortcoming of these single-shot approaches is a decrease in spatial resolution, due to the fact that multiple pixels are merged to extract the phase information.

The reverse projection method^11^ also enables phase retrieval without the need of a stepping procedure. This is achieved by a linear approximation of the stepping curve at its steepest point. However, this entails that the retrieved phase is only correct for small refraction angles, which effectively decreases the dynamic range of the system. To obtain both absorption and phase-contrast information, two interferograms at opposing angles have to be recorded, which requires a full scan over 360 degrees. Furthermore, the dark-field signal cannot be simultaneously obtained by this method. A similar approach^12^ utilizes a linear approximation in the reconstruction step to obtain a combined image containing information both from attenuation and refraction from only one interferogram. However, it is not possible to obtain separate maps of the absorption and refraction of the measured object.

One could imagine to record a full stepping curve without moving the gratings but instead through movement of the imaging setup^13^. Here, the sample is moved over different detector positions instead of performing a translation of the gratings. If the reference phase is not constant over the area of the detector, the same region of the sample is recorded at different fringe phases by this scanning approach. Different fringe phases correspond to different relative positions of the gratings in a traditional phase-stepping approach. Therefore, a stepping curve can be obtained by combining these different areas as illustrated in Fig. 1(b,c). In summary, phase stepping is performed without translating the gratings, but is analogous to that obtained with the standard technique. This fringe-scanning method has been illustrated to work for radiographic scans at synchrotron facilities as well as in a laboratory setup^14^. Recently, a commercial mammography system was converted to a grating interferometry system^15^ using such a fringe-scanning approach. It features multiple commercially available gratings and several line detectors.

In this work, we aim to extend this approach to the tomographic case.

The grating alignment procedure differs for a phase stepping or a fringe scanning measurement. In a phase-stepping configuration the reference phase would ideally be constant over the whole detector area, which is achieved by perfect alignment of the gratings. Here, the sample is recorded at different fringe phases by translating the gratings. In contrast, the scan approach requires the generation of a periodic fringe phase to encode the phase signal in detector positions. To achieve a periodical fringe phase the gratings *G*_1_ and *G*_2_ are misaligned. moiré fringes appear when slightly modifying the inter-grating distance. These are the result of a period mismatch between *G*_1_ and *G*_2_ caused by the beam divergence or in other words the changed magnification. Due to imperfections in the three gratings, the fringes are tilted instead of being perfectly horizontal or vertical. The orientation of the fringes can be adjusted by slightly rotating one of the gratings^16^.

We propose to adopt a helical motion of the tomographic axis to achieve the translation of the sample for fringe scanning. With our method a continuous helical rotation of the sample or the gantry can be achieved, because no stepping of the gratings is needed. In medical CT systems a helical scanning procedure is employed to extend the field of view and reduce measurement time^17^. Presently, there are already theoretical, simulation and first experimental studies on helical scanning procedures for PCCT^18^,^19^,^20^. However, none of these methods eliminate the need for phase stepping for each single projection and, thus, do not allow a continuous sample rotation.

In the following we describe how a scanning-type system similar to the one described by Kottler *et al*.^13^ can be realized by upward motion of the tomographic axis during rotation. A sketch of the employed setup and the proposed helical motion is given in Fig. 1(a).

The minimum number of helical rotations corresponds to the number of phase steps *M* that are ‘planned’ to be recorded for each projection. For each angle, each part of the sample has to be in the field of view at least three times, each at a different fringe phase. This is necessary because at least three data points are needed to extract the three image signals: attenuation, differential phase and dark-field. That means that the pitch *p* has to be lower than 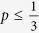. The pitch is defined by the ratio of upward movement per rotation and the detector height. Assuming a tomographic scan over only 180 degrees, two steps can be recorded with one rotation. In this case, the pitch can be as high as 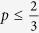. To be able to apply a standard processing algorithm, a full period of the stepping curve has to be sampled at equidistant positions. Therefore, the vertical movement per rotation is dependent on the period of the moiré fringes in the interferogram. After *M* + 1 rotations, the phase of the moiré fringe has to be the same as before the first rotation. This leads to the fact that the number of rotations *M* is chosen to be the same as the number of complete fringes *k* visible in the interferogram. The upward movement per rotation is then given by the active area of the detector divided by *M* + 1. The active area of the detector is the area that is covered by the *k* complete fringes. Alternatively, it is also possible to use just one fringe that spans the whole detector area. Then, the number of rotations and the corresponding upward movement per rotation can be chosen freely. However, fringes can currently not be tuned this way due to inhomogeneous grating structures. In conclusion, with a suitable combination of fringe period and helical movement, a scanning type system for tomography can be realized.

## Results

In this article, we present first experimental results of CT measurements obtained with the proposed helical fringe-scanning method. A tomographic scan of a phantom was carried out using a Talbot-Lau interferometer with a laboratory X-ray tube. Per rotation, *N*_*θ*_ = 600 images with an exposure time of *t* = 1 s each were recorded. Note, that the sample was not rotated continuously but was instead rotated before each exposure (step & shoot). The period of the moiré fringes in vertical direction was tuned to be 36 pixels. This was achieved by a deliberately introduced slight misalignment of the gratings *G*_1_ and *G*_2_. With the purpose of sampling a complete stepping curve, the vertical motion between two images was 0.05 pixels, which results in a vertical movement per rotation of Δ*h* = 30 px. In total, *M* = 6 rotations were recorded for each region of the sample.

**Figure 2:**
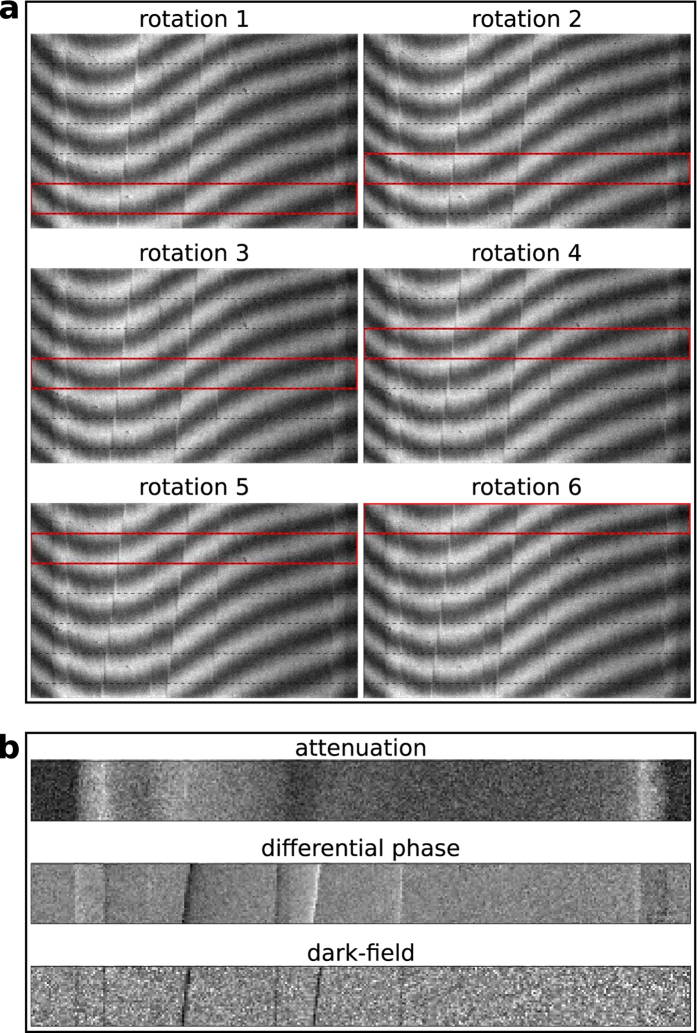
Experimental procedure of helical moiré fringe scanning. Six interferograms (**a**), each recorded at the same angle but at different sample positions because of the helical movement, are combined to obtain a phase-stepping curve for each pixel. The areas marked with a red box are used to obtain a transmission, differential phase and dark-field projection (**b**).

Figure 2(a) illustrates the interferograms with the object in the beam for each of the 6 rotations at the same view angle. The regions of interest indicated by red boxes were combined in one stepping series. Processing in the form of an expectation maximization (EM) algorithm, which is explained in the Methods section, was applied to extract the attenuation, differential phase and dark-field projections as shown by Fig. 2(b). In this manner, a region of 3 mm (30 pixels with 100 μm effective pixel size) of the sample is covered. The total exposure time for each region is *t*_*total*_ = *MN*_*θ*_*t* = 1 h. For each additional rotation (10 minutes) a new region of the sample can be processed, because this region has already been in the field of view *M* − 1 times. All together, the measurement consisted of 13 rotations, which corresponds to a sample coverage of 2.4 cm. Additionally, reference images without the sample were recorded before and after the tomographic scan.

**Figure 3:**
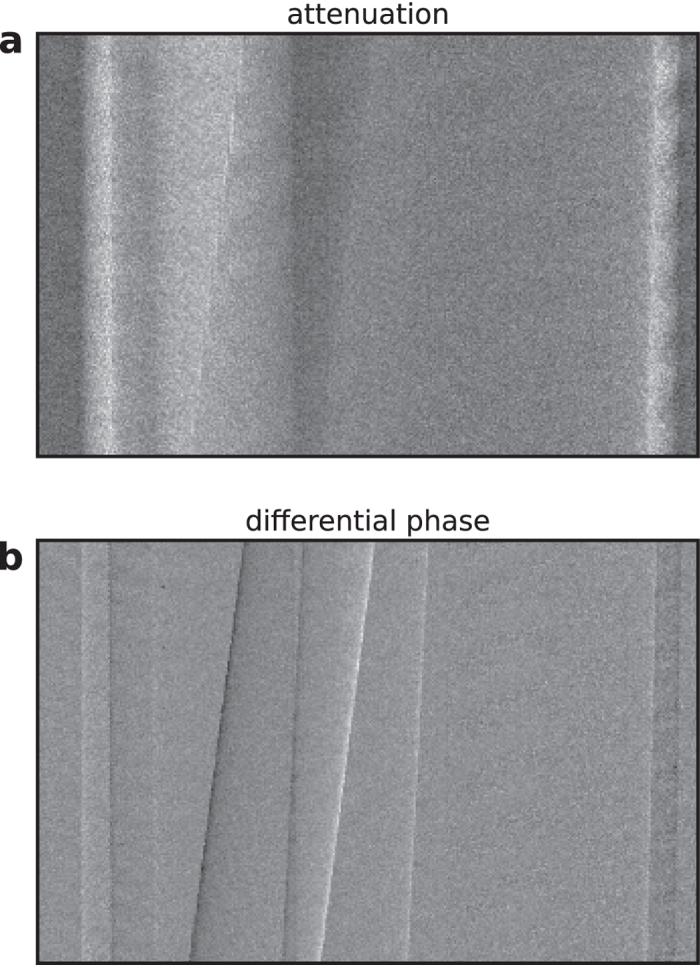
Combined, full-view projections. The projections obtained from the combination of several height steps can be assembled to form a full projection that is even larger (327 pixels × 210 pixels) than the field of view (327 pixels × 195 pixels) of the system.

Figure 3 shows retrieved attenuation and differential phase projections of the measured phantom obtained as described above. The size of the retrieved projections is 365 pixels × 240 pixels, which is larger than the field of view of the detector. In principle, there is no limit on the z-coverage, that is on the vertical size of the sample. Just one additional rotation is needed when the size of the sample increases by Δ*h*. As a result, there are no constraints imposed on the vertical field of view by the gratings and the detector.

As mentioned previously, imperfect and inhomogeneous gratings lead to bend and tilted fringes over the field of view. In our experiment the fringe period is smaller in the left part than in the right part of the interferograms, with the period ranging from 35 to 40 pixels. Therefore, the sampled stepping curve is not of exactly one period in all areas. In these cases, standard processing leads to an error that is dependent on the fringe phase of each pixel in the interferogram, which results in remaining fringes in the projections.

Therefore, a more advanced algorithm was used to extract the three signals from the phase stepping data. This algorithm uses an (EM) approach to correct for variations in the stepping positions (cf. Methods section). Thus, artifacts arising from a non-equidistant sampling of the stepping curve can be avoided. As in the traditional phase-stepping scheme, these artifacts are a result of grating instabilities that lead to wrongly sampled stepping curves. In addition, incompletely sampled stepping curves occur in the helical fringe-scanning method when the fringe period varies over the field of view. This incomplete sampling can result in artifacts in the processed projections. Using the EM algorithm here, these artifacts arising from non-uniform fringe periods can be avoided. Future gratings obtained with improved fabrication processes may be more homogeneous, which will make the alignment procedure less challenging and may make the usage of advanced processing algorithms obsolete.

Artifacts can also be a result of thermal drift of the gratings. The acquisition of the phase steps in helical fringe-scanning PCCT is separated by a full rotation and, thus, by a longer timespan than in a phase-stepping acquisition. For this reason, the scheme is more sensitive to thermal drifts. However, the thermal drift of our setup posed no problem for the acquisition of helical PCCT data. Further, the EM processing scheme would also be able to compensate for these effects and therefore avoid artifacts in the processed projections.

**Figure 4:**
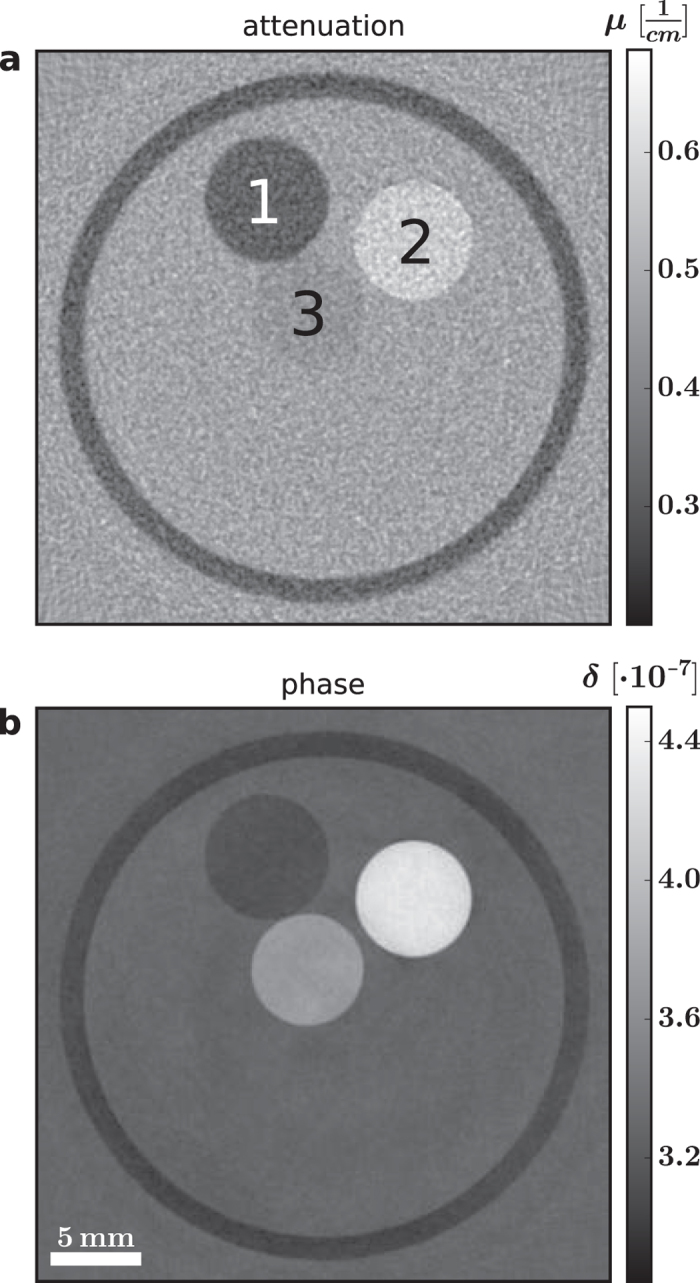
Results of the tomographic reconstruction. The acquired differential phase projections can be utilized to reconstruct the distribution of the refractive index decrement of the measured sample. This figure shows an axial slice of the tomographic reconstruction of the linear attenuation coefficient and the refractive index decrement. The three phantom materials LDPE (1), POM (2) and PMMA (3) are clearly visible.

The processed projections were finally used for a tomographic reconstruction. First, the vertical displacement of the projections due to the helical motion was corrected. Then, filtered backprojection was employed to reconstruct the distribution of the linear attenuation coefficient and the refractive index decrement. A Cone-beam projector, that models the actual geometry of the experimental setup correctly, was used. In the case of the differential phase-contrast projections a Hilbert filter kernel was applied to perform the necessary integration^21^. Figure 4 displays an axial slice of the measured sample in attenuation and phase contrast. The three plastic rods are clearly visible in the tube filled with water.

## Discussion

We have shown a method that – for the first time – enables to perform a phase-contrast CT scan with stationary gratings and that delivers the complete sample information without any spatial interpolation. The essence of this statement is that we are able to extract the three imaging signals for each pixel separately. In contrast, previous methods used several pixels jointly to extract the image signals^8^,^9^,^10^ resulting in an inherent decrease in spatial resolution or the number of pixels in the final image, respectively. Nevertheless, the resolution of our novel acquisition method can be worse compared to a phase-stepping acquisition due to inaccuracies in the experimental implementation.

Additionally, large cone angles lead to inaccuracies in the retrieved signals and a decrease in resolution when using standard reconstruction techniques. However, it is possible to model the correct geometry in novel iterative reconstruction schemes. Recently, methods that do not rely on intermediate phase retrieval have been introduced^22^,^23^. Using these methods, variable geometry for different phase steps resulting from the large cone beam can be considered and correctly modelled. Consequently, data from a helical fringe-scanning acquisition with a cone beam setup can be reconstructed correctly. However, it has not been shown experimentally, yet.

Keep in mind that large samples can also be imaged without the use of large cone angles in a scanning approach like the one demonstrated here, since the FOV in vertical direction is extended beyond the area of the gratings, detector and the X-ray beam. In our case, the cone angle is less than 1 degree and can be approximated by a parallel beam. Therefore, the resolution in the z-axis is not affected even when non-iterative reconstruction is employed. A detailed comparison of the spatial resolution of helical and conventional PCCT may be subject of future work.

With the novel method presented here, some previous limitations of grating-based phase-contrast CT are overcome. This may lead to an easier translation of phase-contrast CT to mainstream applications, possibly including clinical imaging. In particular, we have shown that grating-based phase-contrast tomography can be performed by combining a helical motion of the sample with deliberately induced moiré fringes, thus without the need to translate the gratings. This procedure simultaneously yields conventional attenuation, phase-contrast and dark-field data and enables a continuous rotation, one important requirement for clinical CT systems. In traditional phase-stepping acquisitions, the speed of the stepping motors is a limiting factor in the reduction of the acquisition time. Due to the fact that no stepping procedure is necessary, the acquisition time when using the proposed method is only limited by the exposure time, which enables faster phase-contrast CT scans.

While no true continuous scan was recorded in this work, employing a continuous rotation is straight forward and the same constraints as in absorption CT come into play, where a continuous rotation is standard in medical CT systems. In particular, the exposure time has to be small enough to ensure that there is not too much sample movement at the edges of the sample. Too much movement of the sample during the acquisition of one image results in blurring in the outer parts of the tomographic image. If we had employed a continuous acquisition here, the outer parts of the sample (sample size: 300 pixels, cf. Methods) would have moved *π* × 300 pixels/600 = 1.5 pixels during the acquisition of one image. This is roughly the size of the projection of the source size onto the detector, or in other words, the system point spread function. Therefore, no additional blurring would be expected in this case.

A shorter exposure time per projection, and by that less sample movement during one exposure, reduces the blurring that results from the continuous movement. While reducing the exposure time in a conventional setup can be challenging, there are several approaches to tackle this problem. For one, there is the possibility to use gratings fabricated with thinner, less absorbing wafers to increase the flux at the detector. Furthermore, our X-ray tube has very low flux compared to tubes that are used in a clinical setting. Using a tube of this kind would also enable in a significant reduction of the exposure time. Additionally, the exposure time per projection can be decreased without reducing the total exposure time by increasing the number of acquired projections accordingly. Acquiring e.g. 2400 projections with 0.25 s exposure time instead of 600 projections with 1 s exposure time each, would reduce the blurring by a factor of 4. This is independent of the flux of the tube or other setup parameters. Since we employ a photon-counting detector, this will not change to noise in the tomographic reconstruction^24^.

In contrast to previously introduced methods that allow a continuous rotation, the gratings can be fixed. The need to translate the gratings with a precision of fractions of the grating pitch, which is around 5 μm, is eliminated. With fixed gratings the mechanical stability of the system is less critical. This is especially important considering systems where not the sample but a gantry consisting of source, gratings and detector is rotating^25^. Keep in mind that a precision of 1% of the stepping curve is equivalent to a relative grating displacement of 50 nm.

Instead, in case of the helical fringe scanning approach the sample needs to be translated, but with much less precision, namely to pixel sizes, which range from 50 μm for mammographic systems to over 500 μm for clinical CT systems. In our case, the pixel size (considering magnification) was 100 μm and the fringe period spanned 30 pixels. Thus, a precision of 1% of the stepping curve equals a sample displacement of 30 μm. While it may still be a challenge, this stability requirement is relaxed by three orders of magnitude compared to the conventional phase-stepping approach and can be further relaxed by using larger pixel sizes and larger fringe periods. Also, this sample motion is no different than the one performed in conventional spiral CT systems and may be necessary anyway when objects larger than the area of the gratings or the detector are to be measured. Compared to previously introduced methods that rely on fixed gratings, there is no inherent loss in resolution and the full information (attenuation coefficient, refractive index decrement and scattering strength) can be accessed. This comes with the drawback of a slightly more challenging grating alignment procedure.

In conclusion, we demonstrated a method that allows phase-contrast tomography scans with continuous sample rotation without an inherent loss in resolution. Further, due to the scanning approach the field of view is extended in vertical direction, which enables the imaging of objects larger than the field of view of the system. Lastly, the stability requirements of the imaging system are relaxed due to fixed gratings. Overcoming these limitations is an important step towards industrial and clinical application of grating-based phase-contrast computed tomography.

## Methods

### Experimental Setup

A Talbot-Lau interferometer was used to experimentally test the proposed method. It consists of three gratings *G*_0_, *G*_1_, and *G*_2_ made out of gold with periods of 5.4 μm. The absorption gratings *G*_0_ and *G*_2_ have heights of 60 to 70 μm. The phase grating *G*_1_ was designed to introduce a phase shift of *π* at 27 keV and has a height of 5.2 μm. The setup was installed in a symmetric configuration with inter grating distances of 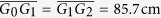. The X-rays are generated by an ENRAF Nonius rotating anode X-ray tube with a Molybdenum target, which is operated at 40 kVp and 70 mA. The projected source size at the position of the detector is roughly 1.5 pixels. A PILATUS II single photon counting detector by *Dectris Ltd*., Switzerland was utilized. It features a field of view of 487 pixels × 195 pixels (horizontal x vertical) with a pixel size of 172 μm × 172 μm. The measured phantom consists of three plastic rods of PMMA (*C*_5_*H*_8_*O*_2_), LDPE (*C*_2_*H*_4_) and POM (*CH*_2_*O*), each with a diameter of approximately 6 mm. They were measured in a tube with a diameter of 3 cm filled with water, which was itself put in a water bath to avoid phase wrapping artifacts^26^,^27^. The sample was mounted at a position where the geometric magnification of the setup is 1.72. Therefore, the effective pixel size at the sample position was 100 μm × 100 μm.

### Expectation-maximization (EM) processing algorithm

We employed an expectation-maximization (EM) algorithm to process the stepping data. This algorithm is able to correct for systematic fluctuations in the stepping positions. It treats the unknown stepping positions as latent or hidden variables and approximates them, together with the image signals.

The intensity curve is described by its linearised form


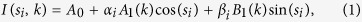


where *k* denotes the pixel index, *s*_*i*_ the stepping positions, and *i* the index of the stepping positions. The three image signal absorption, differential phase and visibility/dark-field can be obtained by evaluating


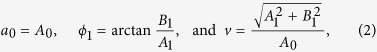


respectively. The parameters *α*_*i*_ and *β*_*i*_ are linked to the the stepping positions by


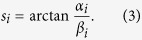


A log-likelihood function can be defined as


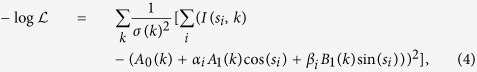


where *σ*(*k*) weights the pixels assuming Gaussian noise.

The EM algorithm consists of two alternating steps. First, using a reasonable starting value for the stepping positions *s*_*i*_ (e.g. equidistant stepping over one period), the parameters *A*_0_, *A*_1_, and *B*_1_ are evaluated (for each pixel independently) using least-squares fitting. Second, with the parameters calculated in the first step, the optimal value for *s*_*i*_ is calculated (for each stepping image separately) by again solving a linear system of equations using least-squares fitting. These two steps are then repeated until the change in stepping positions between consecutive iterations is below a threshold and thus convergence can be assumed.

Note that the the parameters *A*_0_, *A*_1_, and *B*_1_ depend on the pixel number and not on the stepping position, while *α*_*i*_ and *β*_*i*_ only depend on the stepping position. Thus, the deviation from the ideal stepping positions that is recovered by the EM algorithm is constant over the whole projection. Since the fringe periods and thereby also the stepping positions are not constant over the whole projection in our experiment, we divided the projections in regions spanning the whole height of the detector and a width of 30 pixels. Using this algorithm instead of a conventional least-squares fitting of the image signals (i.e. only the first step of the EM algorithm), the fringes in the projections could be greatly reduced which increased the quality of the reconstructions significantly.

## Additional Information

**How to cite this article**: Marschner, M. *et al*. Helical X-ray phase-contrast computed tomography without phase stepping. *Sci. Rep*. **6**, 23953; doi: 10.1038/srep23953 (2016).

## Figures and Tables

**Figure 1:**
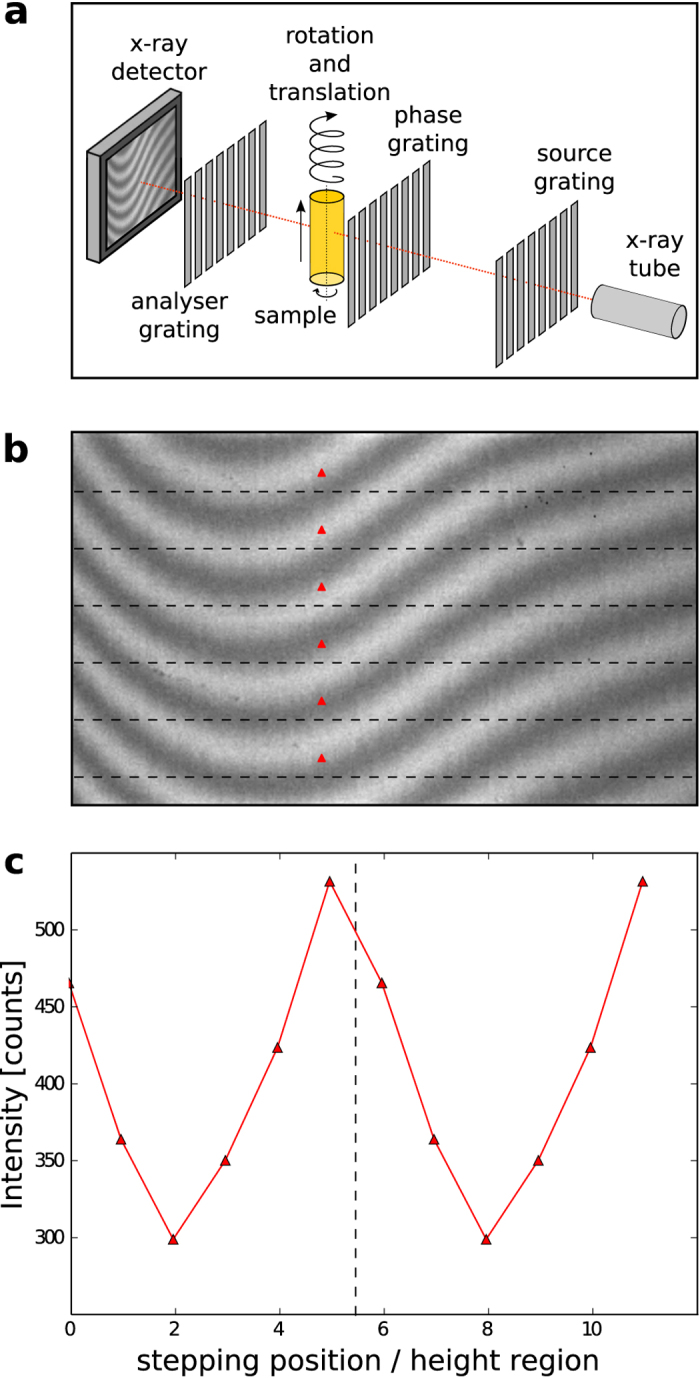
Principle of moiré fringe scanning. A conventional X-ray tube is equipped with a three-grating interferometer for phase-contrast measurements (**a**). The interferogram (**b**) with a size of 327 pixels × 195 pixels shows moiré fringes introduced by a deliberate mismatch of the relative positions of the phase grating and the analyser grating. A stepping curve can be obtained by combining the height regions (327 pixels × 30 pixels), marked by dashed lines. Panel (**c**) shows an exemplary stepping curve that is obtained by using the pixels marked by red triangles.
